# Reviewed article: Alves NS, Silva EN, Melo GBT, Paulino MAS, Tuesta AA. Agenda for COVID-19 and long COVID research priorities in Brazil: results of wide consultation and Delphi consensus in 2022 and 2023. Epidemiol Serv Saude. 2025:34;e20240623

**DOI:** 10.1590/S2237-96222025v34e20240623.b

**Published:** 2025-06-20

**Authors:** Antonio Junior

**Affiliations:** Universidade Federal de Goiás, Internal Medicine

**Table:** 

Questionnaire	Yes	No	Not applicable
Does the manuscript contain new and significant information to justify publication?	✓		
Does the Abstract (Summary) clearly and accurately describe the content of the article?	✓		
Is the problem significant and concisely stated?	✓		
Are the methods described comprehensively?	✓		
Are the interpretations and conclusions justified by the results?	✓		
Is adequate reference made to other work in the field?	✓		

**Table:** 

Rating	Excellent	Good	Average	Below average	Poor
Interest		✓			
Quality			✓		
Originality			✓		
Overall			✓		

## Manuscript Structure

Length of article is: Adequate

Number of tables is: Adequate

Number of figures is: Adequate

Please state any conflict(s) of interest that you have in relation to the review of this paper (state “none” if this is not applicable): None

## Comments

Resumo:

O estudo propõe uma agenda de prioridades de pesquisa sobre COVID-19 e pós-COVID no Brasil, utilizando uma metodologia Delphi para alcançar consenso entre especialistas. Essa agenda é projetada para orientar financiamentos futuros e direcionar esforços de pesquisa, destacando a necessidade de abordar as sequelas da COVID-19, com um enfoque especial nas crianças.

Comentários Gerais:

• Pontos Fortes:

o Relevância Temática: A escolha do tema é extremamente relevante para o contexto atual e futuro do Brasil, considerando os impactos prolongados da COVID-19.

o Metodologia Rigorosa: O uso do método Delphi para formar um consenso entre uma variedade de stakeholders é uma abordagem robusta e apropriada para o objetivo do estudo.

• Pontos Fracos:

o Representatividade da Amostra: Embora o estudo tenha envolvido uma variedade de participantes, a representatividade de todas as regiões e grupos sociais poderia ser melhor detalhada para reforçar a abrangência das conclusões.

o Detalhamento dos Dados: Falta uma discussão mais profunda sobre como as linhas de pesquisa foram priorizadas individualmente e o critério específico para inclusão ou exclusão nas rodadas subsequentes do Delphi.

Comentários Específicos:

• Introdução e Justificativa (Linhas 10-35):

o A introdução estabelece bem o contexto e a importância do estudo, mas poderia expandir a discussão sobre as especificidades do impacto da COVID-19 no Brasil, que justificam a necessidade de uma agenda de pesquisa dedicada.

• Metodologia (Linhas 65-85):

o A metodologia é descrita com detalhes suficientes, porém, a explicação sobre a seleção dos participantes para o estudo Delphi e a justificativa para o número de rodadas poderia ser mais explícita para evitar qualquer viés percebido.

• Resultados (Linhas 120-140):

o Os resultados são apresentados de maneira clara. No entanto, seria útil incluir uma análise quantitativa ou qualitativa mais detalhada sobre como as diferentes prioridades emergiram das discussões.

• Discussão (Linhas 180-200):

o A discussão correlaciona bem os resultados com a literatura existente, mas poderia se beneficiar de uma crítica mais aprofundada sobre como essas prioridades de pesquisa podem ser efetivamente implementadas dentro do sistema de saúde brasileiro.

Adequação Ética e Metodológica:

• O estudo segue normas éticas apropriadas, com clara descrição do consentimento informado. A metodologia é adequada para o tipo de pesquisa, mas recomenda-se uma maior transparência no processo de análise de dados.

